# Tourniquet self-application assessment in cold weather conditions

**DOI:** 10.1186/s12873-023-00871-1

**Published:** 2023-08-31

**Authors:** Carlos Yánez Benítez, Teófilo Lorente-Aznar, Idurre Labaka, Marcelo A. F. Ribeiro Jr, Yosu Viteri, Koji Morishita, Marta Baselga, Antonio Güemes

**Affiliations:** 1 General and GI Surgery Department, San Jorge University Hospital, SALUD, Avenida Martínez de Velasco, 36, Huesca, 22004 Spain; 2 Jaca Health Center, SALUD, Paseo de la Constitución, 8, Jaca, Huesca, 22700 Spain; 3 Emergency Medicine, Donostia University Hospital, C/ Begiristain Doktorea Pasealekua, Donostia, Spain; 4 Division of Trauma, Critical Care, and Acute Care Surgery, Sheikh Shakhbout Medical City, Khalifa University, Abu Dhabi, United Arab Emirates; 5 Emergency Department, Torrelodones University Hospital, Avenida Castillo Olivares, s/n, Madrid, 28250 Spain; 6 Department of Acute Critical Care and Disaster Medicine, Tokyo Medical and Dental University Hospital of Medicine, Tokyo, Japan; 7 Clinical and Experimental Research Group, Institute for Health Research of Aragón, C/ de San Juan Bosco, 13, Zaragoza, 50009 Spain; 8 Department of General Surgery, Lozano Blesa University Hospital, Avenida San Juan Bosco, 15, Zaragoza, 50009 Spain

**Keywords:** Training, Trauma, Vascular, Tourniquet

## Abstract

**Background:**

Our study aimed to assess the ability of nonmedical civilians to self-apply extremity tourniquets in cold weather conditions while wearing insulating technical clothing after receiving basic training.

**Methods:**

A field study was conducted among 37 voluntary participants of an expedition party to the Spanish Antarctic base. The researchers assessed the participant’s ability to self-apply five commercial extremity tourniquets (CAT, OMNA, RMT, SWAT-T, and RATS) over cold-weather clothing and their achieved effectiveness for vascular occlusion. Upper extremity self-application was performed with a single-handed technique (OHT), and lower extremity applying a two-handed technique (THT). Perceptions of self-application ease mean values ± standard deviation (SD) were compared by applying a 5% statistical significance threshold. Frequency count determined tourniquet preference.

**Results:**

All the tested ETs, except the SWAT-T, were properly self-applied with an OHT, resulting in effective vascular occlusion in the upper extremity. The five devices tested were self-applied correctly in the lower extremities using THT. The ratcheting marine-designed OMNA ranked the highest for application easiness on both the upper and lower extremities, and the windlass CAT model was the preferred device by most participants.

**Conclusions:**

Civilian extremity tourniquet self-application on both upper and lower extremities can be accomplished in cold weather conditions despite using cold-weather gloves and technical clothing after receiving brief training. The ratcheting marine-designed OMNA ranked the highest for application ease, and the windlass CAT model was the preferred device.

**Supplementary Information:**

The online version contains supplementary material available at 10.1186/s12873-023-00871-1.

## Background

The conflict in Ukraine in the cold February of 2022 has brought attention to the issue of extremity injuries among civilians in war. These injuries are common in modern warfare and often lead to exsanguination, the leading cause of death [1]. According to the United Nations High Commissioner for Human Rights, from the start of Russia’s attack on Ukraine until June 6th, 5,141 civilians have been injured [2]. Ukraine experiences mean winter temperatures ranging from − 4.8ºC to 2ºC, and winter sports athletes are also at risk for extremity injuries with associated vascular injuries and increased mortality [3–6]. Studies have shown that extremity tourniquet (ETs) can be an effective temporary measure for controlling life-threatening bleeding [7–9]. However, wound exposure and direct ET application may lead to hypothermia in isolated cold-weather regions. To address this issue, ET self-application (ET-SA) over winter clothing may be an effective temporary method for controlling extremity bleeding in cold weather scenarios [10, 11].

The Committee for Tactical Combat Casualty Care (CoTCCC) has studied ET models for military prehospital use [12]. Nonetheless, the assessment of ET-SA by civilians with limited training in cold weather needs to be improved. ET-SA can be a crucial skill taught to civilians in battlefield situations where high-velocity missiles and blast injuries can cause severe extremity injuries. It’s also of utmost importance for those who engage in winter sports or have outdoor winter-related jobs to acquaint themselves with ES-SA, which can make all the difference between survival and tragedy.

ET designs, apparently simple to use, can become challenging to self-apply in icy conditions [13]. Furthermore, effective vascular occlusion has not been determined when ETs are self-applied (SA) over cold-weather clothing after brief training. The researchers designed a field study at the Spanish Antarctic base (SAB) “Juan Carlos I” to address these issues. The primary objective was to determine participants’ capacity to self-apply ETs in cold weather after receiving a one-day training session. Secondary objectives were to determine if such ETs were effective for vascular occlusion in the upper extremity when used over cold-weather clothing, ET-SA easiness, pain tolerance, and device preference. Despite cold conditions, we hypothesized that the trained civilian participants could properly self-apply ETs to the upper and lower extremities. Also, ETs would be effective for extremity vascular occlusion in the upper extremity if SA over cold-weather technical clothing.

## Methods

A group of 80 individuals went on an expedition to the SAB, and 37 participated in an ET-SA assessment. The assessment took three phases: a one-day lecture and hands-on training module, an outdoors self-application assessment, and a post-activity survey. During the assessment, participants used five commercially available ETs, which included the most common designs: windlass, ratcheting, and elastic. The windlass model used was the Combat Application Tourniquet Gen − 7 (CAT), while the ratcheting models included the Ratcheting Medical Tourniquet (RMT) and OMNA Marine Tourniquet (OMNA). The elastic models used were the Stretch-Wrap-And-Tuck Tourniquet (SWAT-T) and the Rapid Application Tourniquet System Gen − 2 (RATS).

All the individuals who participated in the study were civilians without military backgrounds and had no experience applying tourniquets to themselves. Before leaving for the SAB, everyone underwent a medical examination, and none had any history of clotting or vascular disease. The Spanish polar research crew’s survival program included study-related activities. All the participants willingly volunteered for the study, gave informed consent, and agreed to the public use and publication of any recorded images.

The training module focused on compressible hemorrhage, which consisted of a 4-hour lecture followed by a 4-hour hands-on practice with all five devices. The field study was performed on Livingston Islands’ glaciers’ surface after being outdoors for at least 6 h. To calculate the daily mean temperatures, the maximum and minimum temperatures were added together for 24 h and then divided by two. The range of temperatures recorded during the study was between − 2ºC and 3ºC [14]. However, since the field study was conducted on several glaciers, including Johnsons’, Hurd, Huntress, Perunika, Kamchiya, and Verila, the authors used wind chill tables to estimate the effects of cold wind on the exposed body surface. This corresponded to a perceived temperature of -10ºC to -20ºC [15, 16]. Party members on each glacier day trip comprised five persons (2 mountain guides, two glacier researchers, and the study coordinating physician). On each trip, only two-party members performed the ET-SA exercise, first on the upper extremity, followed by the lower one. A total of 18 one-day trips were necessary to complete the field phase. Participants performed the exercise with their dominant hand with the five devices in a randomized order to avoid bias and preference. In total, ten tourniquet applications were performed by everyone, with five different ETs on the upper and lower extremity. The expedition study coordinator and physician supervised the activity and assessed adequate self-application and on-site effectiveness.

The participants wore the same technical clothing, including three layers to keep them warm and dry. They had a polyester or polypropylene base layer, a puffy middle layer, and an outer shell that protected them from wind and water. ET-SA was performed while wearing cold weather-protecting fingered gloves, both in the upper extremity, applying a one-handed technique (OHT) and a two-handed technique (THT) in the lower one. The tourniquets were consistently applied to the non-dominant upper extremity and the right lower extremity without alternation. After removing gloves, a 5-minute gap was established between each tourniquet application to check pulse oximetry. During verifying vascular occlusion, participants remained ungloved for less than a minute. They were then allowed 5 min to recover and wear gloves between applications.

Three aspects were required to consider SA adequate: ET positioning (mid-brachium for the upper extremity and mid-thigh for the lower extremity), device tightening, and securing it in a compressed and locked position, all without help. After ET-SA in the upper extremity, the physician assessed its effectiveness by pulse checks, and using a Choice Med Fingertip MD300C2 pulse oximetry sensor (PO). This portable device displayed oxygen saturation (SpO2), pulse rate (PR), a pulse bar graph, and the SpO2% waveform on a dual-color OLED display. The measurements were taken after removing the glove used for self-application, allowing the physician to check the pulse, and read the OLED. Due to the conditions of the field study, researchers did not perform pulse checks, PO, or SpO2% waveform registry on the lower extremity.

After completing the field phase, an anonymous questionnaire was issued to each participant to determine the easiness of SA on both the upper and lower extremities. Ease of SA and pain tolerance level were measured using a 10-point Likert scale (one for the least easy or least tolerable, and ten for the easiest or most tolerable). Finally, it questioned tourniquet preference, asking participants to choose only one of the five devices tested. Researchers applied descriptive statistics for the demographical data. The mean values ± standard deviation (SD) of the ET’s -SA easiness and tolerance were compared using a one-way repeated measures ANOVA and a Bonferroni post hoc pair-wise comparison test, with a 5% statistical significance threshold. Statistical analyses were performed using the JAMOVI 1.6.3 open statistical platform [17]. and IBM-SPSS Statistics 10.1.

## Results

Thirty-seven (n = 37) volunteers participated and completed the study, 33 (89%) males and 4 (11%) females, ages ranging from 23 to 59, with a mean age of 42 years. None of the participants could SA the SWAT-T model on their upper extremities using a OHT but could use a THT in their lower extremity. The other four devices were successfully SA, both in their upper and lower extremity, following the OHT and THT. Researchers identified no SA disparity between males and females on the upper or lower extremities.

**Fig. 1:**
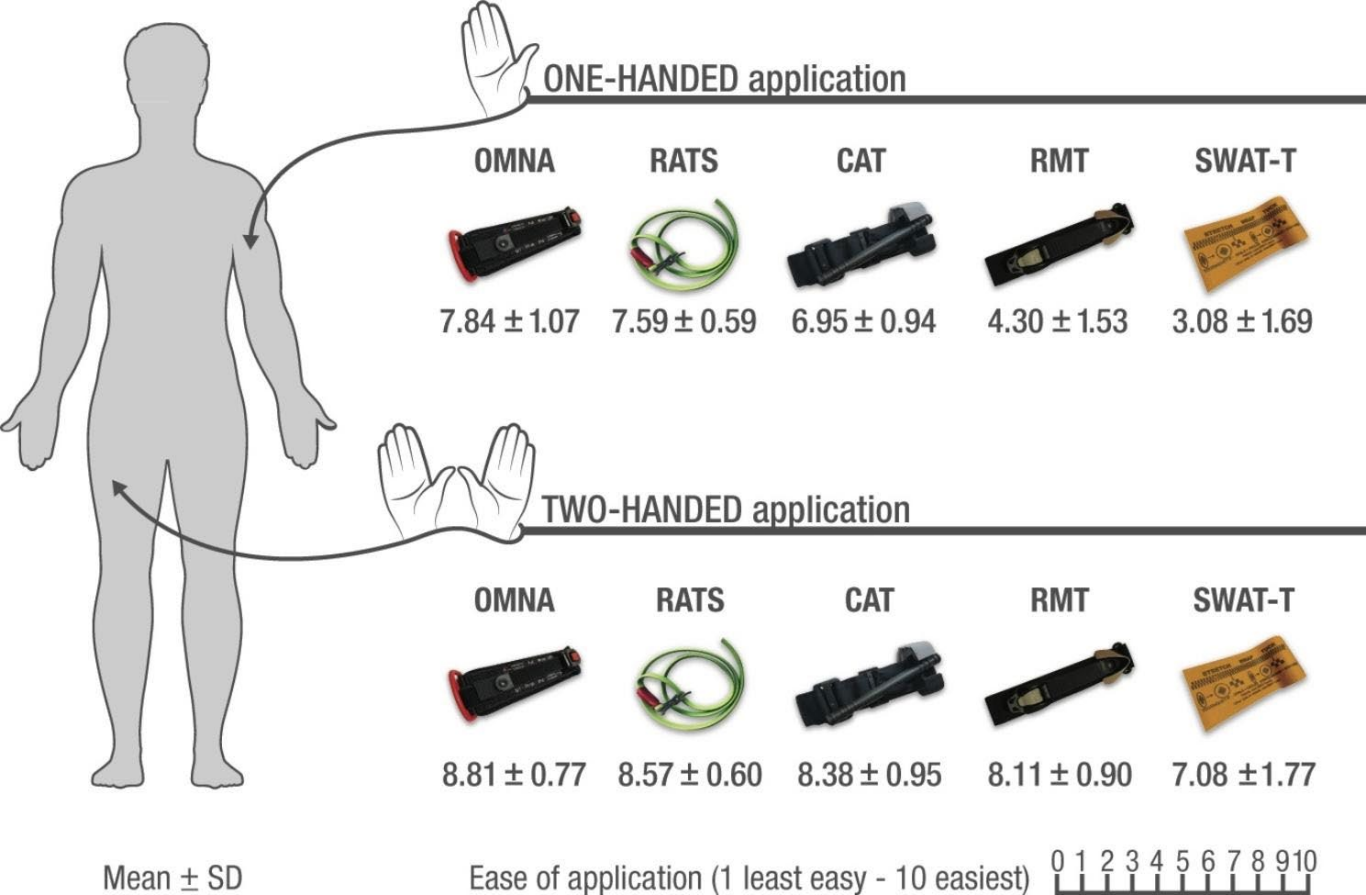
Easiness mean values for ET self-application on an upper non-dominant extremity (OHT) and lower dominant extremity (THT) for the different devices tested. (ET: Extremity Tourniquet, OHT: One-handed technique, THT: Two-handed technique, OMNA: OMNA Marine Tourniquet, Rapid Application Tourniquet Gen − 2, CAT: Combat Application Tourniquet Gen − 7, RMT: Ratcheting Medical Tourniquet, SWAT-T: Stretch-Wrap-And-Tuck Tourniquet). One-handed ease of self-application with CAT, RATS and OMNA is statistically better than with RMT and SWAT (repeated measures ANOVA with Bonferroni post hoc comparison, p < 0.01). In the two-handed self-application, all show to be statistically easier to place than SWAT (p < 0.001)

Upper extremity effective vascular occlusion was confirmed in the four adequately self-applied devices: CAT (Gen − 7), RMT, OMNA, and RATS. The one-way repeated ANOVA of the mean values ± SD for the perception of one-handed self-application easiness comparing the five devices on the upper extremity revealed a significant difference (Wilks’ Lambda = 0.03, F (4.33) = 230.6, P < 0.001). Post hoc tests using the Bonferroni pair-wise comparison test pointed to the ratcheting OMNA as the easiest, followed by the elastic RATS (Gen-2) and the windlass CAT (Gen-7). **(**Fig. 1**)** The ratcheting RMT and the elastic SWAT-T models were ranked as more challenging to self-apply than the others. Their difference compared to the rest was statistically significant (P < 0.05).

The one-way repeated ANOVA for THT self-application easiness was also calculated, finding a significant difference among the mean values (Wilks’ Lambda = 0.50, F (4.33) = 8.18, P < 0.001). The participants reported the OMNA as the easiest to self-apply, followed by the RATS (Gen-2), the CAT (Gen-7), the RMT, and the SWAT-T. **(**Fig. 1**)** The only one that resulted as less easy than the rest, with a statistically significant difference, was the SWAT-T (Bonferroni pair-wise comparison test P < 0.05).

**Fig. 2:**
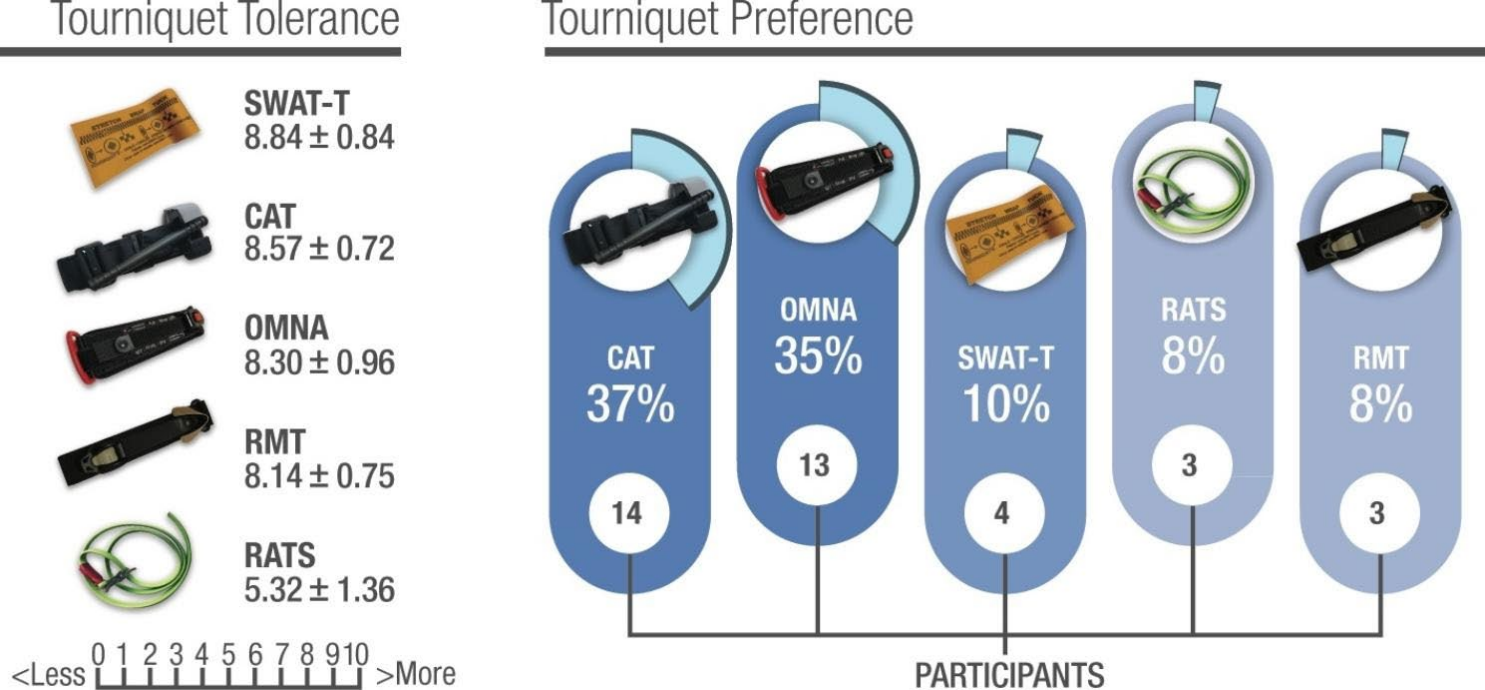
Participants’ ET preference and ET tolerance mean values for the devices tested while wearing cold-weather gloves and technical clothing. (ET: Extremity Tourniquet, OMNA: OMNA Marine Tourniquet, Rapid Application Tourniquet Gen − 2, CAT: Combat Application Tourniquet Gen − 7, RMT: Ratcheting Medical Tourniquet, SWAT-T: Stretch-Wrap-And-Tuck Tourniquet). The tolerance of the self-applied CAT and SWAT is higher than the rest (repeated measures ANOVA with Bonferroni post hoc comparison, p < 0.01), and that of the RATS is lower than all the others (p < 0.001). The preference for CAT and OMNA is statistically higher than the rest (p < 0.01)

Results of mean values ± SD for overall tourniquet pain tolerance revealed that most devices used were well tolerated. The SWAT was valued as the best, followed by the CAT (Gen-7), the OMNA, and the RMT. **(**Fig. 2**)** However, the tolerance assessment for the SWAT was exclusively for the lower extremities since none of the participants could properly self-apply it around their upper extremities. The elastic RATS (Gen-2) model was ranked as the least tolerable (5.3 ± 1.4), statistically different from the rest (Wilks’ Lambda = 0.13, F (4.33) = 54.4, P < 0.001, Bonferroni pair-wise comparison test P < 0.05). The preferred commercial device among the five tested was the CAT (Gen-7), followed by the OMNA and the SWAT-T. **(**Fig. 2**)**

## Discussion

Our study intended to verify the ability of pre-trained civilians to self-apply ETs in cold weather conditions while using cold-weather clothing and gloves. Having skills in ET-SA can be crucial for both military personnel and civilians in cold weather situations, whether hostile or non-hostile. It’s important to undergo thorough training that covers theoretical principles, hands-on practice, and real-time performance feedback with various ET models to ensure the best possible outcomes in the field. Various authors report training for military and civilian personnel [18–21]. However, their activities are limited to certain ET models or mechanisms in controlled laboratory conditions, which may not sufficiently prepare trainees to use models that are commercially available [22]. To improve the readiness and performance of the participants, the authors dedicated eight hours to concentrated training, utilizing five distinct ET models, and providing immediate feedback during the practical exercise. For a more authentic assessment experience, the authors emphasized performing the field exercise in a cold and isolated location after performing research activities on the glaciers for a minimum of 6 h, averaging between − 2ºC and 3ºC. These conditions are prevalent in regions of the northern hemisphere, affecting mountain rescue teams, workers who spend time outdoors in winter, and winter sports enthusiasts across the globe. Even though the conditions were challenging, all participants could properly apply four out of the five tested devices on their upper limbs using OHT. Additionally, every participant could perform a THT with each device on their lower limbs without issues. The finding is considered relevant by the authors, confirming the participants’ capacity to SA most of the ETs tested with limited but specific one-day training, despite the lack of previous expertise, the cold temperatures, the use of insulating gloves, or the bulkiness of their technical clothing. When applying a limb tourniquet over technical clothing, it’s essential to consider the type of material of the ET and the clothing. The type of fabric used can significantly affect the tourniquet’s redirecting buckles, compressibility, and surface interaction. The clothing you wear can significantly impact the effectiveness of the limb tourniquet [23]. Some authors have reported that windlass tourniquet models can be effective when used over chemical, biological, radiological, or nuclear (CBRN) suits [24, 25] and on military winter clothing using a Hapmed ^TM^ Tourniquet Trainer. However, it should be noted that the winter clothing studies were conducted under specific room temperature conditions and with experienced providers who did not use cold weather gloves when applying the tourniquets [10].

The authors consider the distinction between cold weather conditions and the windchill effect experienced outdoors during our research to be significant. Several studies document the repercussions on human response when exposed to temperatures ranging from − 20º to 10ºC, describing vigilance impairment and slower reaction time [26, 27]. The distraction theory explains how severe reductions in ambient temperature disrupt focus and negatively impact cognitive function [28, 29]. Additionally, prolonged cold exposure and reduced skin temperature hamper manual agility, muscular strength, and tactile sensation, all essential for ET-SA [30–33]. Even with proper training, ET-SA can prove challenging due to the frigid weather and specialized clothing required for the study [13]. These factors can complicate even the simplest of tasks. Based on our findings, it seems that the participants could successfully apply most of the ETs themselves, even in the cold and windy conditions of the glacier. Only the SWAT-T self-application resulted in unachievable with the OHT on an upper extremity. The authors believe this finding is more consistent with device design rather than explained by the weather conditions. The SWAT’s elastic strap and lack of auto-locking buckle design require additional training, which results in unachievable with gloved hands in the OHT. All the other assessed ETs were successfully self-applied single-handed using gloves.

Based on our study, it is evident that the strap routing design, pressure generating mechanism, and securing system design are the three key factors that contribute to the ease of use of the ET-SA. Furthermore, our findings indicate that using a two-handed technique for self-application is significantly simpler than using a one-handed technique. This observation partially explains the lower rankings of one-handed self-application on all devices. While the THT had more consistent results, other factors affect the ease of application, such as device design (elastic vs. non-elastic), strap thickness and material, buckle design, and tightening system. Our study found that the OMNA, RATS, and CAT devices were equally easy to self-apply using one hand. These devices had an auto-locking system that required minimal effort and a simple strap mechanism. The CAT and OMNA had a smooth-running strap at the buckle and an easy locking system, making them easy to apply single-handedly. Although the RATS lacks a redirecting buckle, its cleat design allows easy self-application using the strap. However, the ratcheting RMT and elastic SWAT were more challenging to self-apply with a OHT. The RMT strap system often got stuck at the redirecting buckle and did not move smoothly when self-applied, especially with an OHT. The elastic design of SWATs requires special skills to ensure proper traction and precision during each consecutive wrap and the ability to tuck the end under the last wrap for effective self-application. These skills require extra training and can be challenging with a gloved one-handed technique.

Even though the CoTCCC does not recommend some of the ET, [10] the authors included them because some are commonly used in civilian settings. Several of these ET models have proven effective in cadaver-perfused model experiments [34]. Our results show that upper extremity vascular occlusion while wearing multilayered cold-weather clothing seems effectively achieved based on pulse checks and PO registries. Focusing on the assessment method for determining the effectiveness of upper extremity vascular occlusion, the authors acknowledge that PO assessment may be considered suboptimal and, like most other experts, would have preferred a Doppler register [35, 36]. However, the team’s equipment carrying capacity and the field study conditions made impracticable Doppler use in our study. Furthermore, recent studies comparing handheld Doppler devices with PO waveform displays found that the correlation between the registries of both devices was very high for upper extremity determinations [37]. These studies suggest that though Doppler is the gold standard, portable PO devices can accurately determine upper extremity blood flow in a healthy population [38]. PO can result in inaccuracy due to significant levels of dysfunctional hemoglobin, use of intravascular dyes, high ambient light, movement of the extremities, severe peripheral vasoconstriction, severe anemia, hypothermia, and low perfusion states [39]. It has been reported that finger-tip pulse oximetry SpO2 readings can be affected by temperatures below 15ºC. This is due to temperature-dependent arteriovenous shunts in the periphery, which can cause a change in venous oxygen saturation [40]. The authors acknowledge these limitations and reduce their influence by keeping the hand warm and fully gloved during the ET-SA, removing the glove briefly to perform pulse checks and PO detection. Additionally, the Choice Med Fingertip MD300C2 PO used by the authors has a pulse bar indicator that signals when SpO2 and pulse rate value are potentially incorrect, and the physician supervising the activity has vast experience using the device and performing pulse checks. Other authors correlate the accuracy of potable PO registries with arterial blood analysis in actual mountain conditions of altitude (2100 m) and cold weather, finding that portable pulse oximeters correlate well with arterial blood analysis [41]. Our findings, based on pulse rate (PR), pulse bar graph, and SpO2% waveform, suggest that vascular occlusion can be successfully achieved after self-application on the upper extremity with four of the five tourniquets tested, despite the bulkiness of the cold weather gear. The authors consider this result relevant since, to our knowledge, no previous studies have reported such findings with ETs self-applied over three layers of cold-weather clothing in realistic outdoor conditions.

Participants’ tolerance perception was assessed to determine the pain level experienced when the different ET models were applied. Several studies confirm that the wider the strap, the lower the pressure needed for vascular occlusion [42, 43]. This translates to lower skin pressure and less pain, and in our study, all the other four devices (SWAT-T, OMNA, CAT, and RMT) have a wider strap than the RATS. Those four devices were rated above eight on a one-to-ten scale, where ten was the most tolerable. The mean value assigned to the RATS model (5.3 ± 1.4) resulted in statistically significant (P < 0,01) when compared with that of the rest, which is attributable to the device’s slimmer design. When applied, narrow ETs create more soft tissue and nerve injuries, generating more pain, and the thin strap tends to make a pinching effect despite being applied over technical clothing. Another issue with the RATS model that could generate discomfort is the strap securing metal cleat, potentially resulting in painful pressure over the skin when fully fixed. The ET tested with the widest strap (10.4 cm), the SWAT-T, had the highest tolerance (8.8 ± 0.8). Its design is free of buckles and sharp corners; however, this assessment was only appropriate in the lower extremity since it could not be self-applied in the upper extremity, and consequently, vascular occlusion could not be established. Because of that, the authors do not consider this result fit for tolerance measurements even if it ranked as the most tolerable. Finally, the participants’ ET preference was based on an individual comprehensive conception of adequacy, tolerance, and application easiness under the harsh weather conditions encountered during the study. The results showed that the CAT, closely followed by the OMNA, was the preferred device, considering their overall performance and application easiness.

## Limitations

It is imperative to acknowledge the existing limitations, including the nonexistence of an assessment regarding the effectiveness of tourniquets on the lower extremities, the subjectivity involved in evaluating the ease of their application, and personal preference regarding tourniquets. Due to the primarily male population, our study’s findings could not make any conclusions regarding gender. For precise evaluation of efficient vascular occlusion on the upper extremity, forthcoming studies must integrate handheld Doppler devices. This will unequivocally confirm the efficacy of ET when self-applied over thick, cold-weather clothing without any disruption to cold temperatures or cold-induced peripheral vasoconstriction. Moreover, it is imperative for forthcoming studies to thoroughly investigate whether the utilization of the dominant or non-dominant hand significantly impacts the outcome of OHT, while also scrutinizing any inconsistencies in device usage amongst right-handed and left-handed individuals. Furthermore, a comprehensive evaluation of diverse cold-weather glove designs is crucial in determining the most optimal options for ET-SA in harsh icy environments. Moreover, further research ought to be conducted to determine the optimal level of ET application for both the upper and lower extremities. Additionally, the performance of ETs after prolonged exposure to freezing temperatures must be evaluated.

## Conclusion

Even in cold weather conditions and while wearing cold-weather gloves and technical clothing, civilians can effectively apply tourniquets to their upper and lower extremities with proper training. All the tested ETs, except the SWAT-T, were properly self-applied with an OHT, resulting in effective vascular occlusion in the upper extremity. The five devices tested were self-applied correctly in the lower extremities using THT. According to the participants, the OMNA, a marine-designed ratcheting ET was the easiest to self-apply on both the upper and lower extremities. Additionally, most participants preferred the CAT model windlass device.

### Electronic supplementary material

Below is the link to the electronic supplementary material.

Supplementary Material 1

Supplementary Material 2

## Data Availability

This published article and its supplementary information files include the central database supporting the study findings.

## Abbreviations

UN: United Nations
ET: Extremity tourniquet
ET-SA: Extremity tourniquet self-application
CoTCCC: Committee for Tactical Combat Casualty Care
SA: Self-applied
SAB: Spanish Antarctic base
CAT: Combat Application Tourniquet Gen − 7
RMT: Ratcheting Medical Tourniquet
OMNA: (OMNA Marine Tourniquet) Tourniquet (and the
SWAT-T: Stretch-Wrap-And-Tuck
RATS: Rapid Application Tourniquet System Gen- 2
OHT: One-handed technique
THT: Two-handed technique
PO: Pulse oximetry
SpO2: Oxygen saturation
PR: Pulse rate

## References

[1] Gawande A. (2014) Casualties of war–military care for the wounded from Iraq and Afghanistan. N Engl J Med. Dec 9;351(24):2471-5. 10.1056/NEJMp048317. PMID: 15590948. 10.1056/NEJMp048317 15590948

[2] United Nations Human Rights. (2022) Ukraine: civilian casualty update. June 7th, UN Human Rights Monitoring Mission in Ukraine (HRMMU). https://ukraine.un.org/sites/default/files/2022-06/Ukraine%20-%20civilian%20casualty%20update%20as%20of%2024.00%206%20June%202022%20ENG.pdf.

[3] Hohlrieder M, Kroesslhuber F, Voelckel W (2010). Experience with helicopter rescue missions for crevasse accidents. High Alt Med Biol.

[4] Pasquier M, Taffé P, Kottmann A (2014). Epidemiology and mortality of glacier crevasse accidents. Injury.

[5] Eun JC, Bronsert M, Hansen K (2015). Vascular injury is associated with increased mortality in winter sports trauma. Ann Vasc Surg Jan.

[6] Maurer PC. (1975) Gefässverletzungen beim Skisport [Vascular injuries during skiing]. Fortschr Med. Jan 23;93(3):91 – 3. German. PMID: 1126672. 1126672

[7] Goodwin T, Moore KN, Pasley JD (2019). From the battlefield to main street: Tourniquet acceptance, use, and translation from the military to civilian settings. J Trauma Acute Care Surg Jul.

[8] Inaba K, Siboni S, Resnick S (2015). Tourniquet use for civilian extremity trauma. J Trauma Acute Care Surg.

[9] Teixeira PGR, Brown CVR, Emigh B (2018). Civilian Prehospital Tourniquet Use is Associated with Improved Survival in patients with Peripheral Vascular Injury. J Am Coll Surg May.

[10] Lechner R, Beres Y, Oberst A (2023). Analysis of tourniquet pressure over military winter clothing and a short review of combat casualty care in cold weather warfare. Int J Circumpolar Health.

[11] Norheim AJ, Rannestad B, Howes R (2022). Abstracts from the Cold Weather Operations Conference 2021. Int J Circumpolar Health.

[12] Montgomery HR, Hammesfahr R, Fisher AD et al. (2019) Recommended Limb Tourniquets in Tactical Combat Casualty Care. Journal of special operations medicine: a peer reviewed journal for SOF medical professionals. Jan 1;19(4):27–50. 10.55460/HQDV-7SXN 31910470

[13] Brodin W. The effect of cold exposure on tourniquet application ability: the effect of cold hand-skin temperatures on medical laypeople’s ability to apply a tourniquet. Master’s thesis. Linköping; 2022.

[14] Pedrero RA. Temperatura del aire en las islas Livingston y Decepción, Antártida, en el periodo 2006–2021, y análisis de las tendencias de evolución. Universidad de Alcalá; 2022.

[15] Castellani JW, Tipton MJ (2015). Cold stress Effects on exposure tolerance and Exercise Performance. Compr Physiol.

[16] Lankford HV, Fox LR (2021). The Wind-Chill Index. Wilderness Environ Med.

[17] The jamovi project. (2020). *jamovi* (Version 1.2) [Computer Software]. Retrieved from https://www.jamovi.org.

[18] Ross EM, Mapp JG, Redman TT (2018). The Tourniquet gap: a pilot study of the Intuitive Placement of three Tourniquet types by Laypersons. J Emerg Med Mar.

[19] Yánez Benítez C, Ribeiro MAF Jr, Khan M et al. (2021) Extremity Tourniquet Training at High Seas. World J Surg. 2021;45(8):2408–2414. 10.1007/s00268-021-06149-6. Epub 2021 Apr 30. PMID: 33939010. 10.1007/s00268-021-06149-6 33939010

[20] Savage E, Pannell D, Payne E, O’Leary T, Tien H (2013). Re-evaluating the field tourniquet for the Canadian Forces. Mil Med.

[21] Martinez T, Duron S, Schaal JV, Baudoin Y (2018). Tourniquet Training Program assessed by a new performance score. Prehosp Disaster Med.

[22] McCarty JC, Hashmi ZG, Herrera-Escobar JP et al. (2019) Effectiveness of the American College of Surgeons Bleeding Control Basic Training Among Laypeople Applying Different Tourniquet Types: A Randomized Clinical Trial. JAMA Surg. Oct 1;154(10):923–929. 10.1001/jamasurg.2019.2275. PMID: 31339533; PMCID: PMC6659166. 10.1001/jamasurg.2019.2275 PMC6659166 31339533

[23] Wall PL, Buising CM, Hingtgen E (2020). Clothing effects on limb tourniquet application. J Spec Oper Med.

[24] Peponis T, Ramly E, Roth KA (2016). Tourniquet effectiveness when placed over the joint service lightweight, integrated suit technology. J Spec Oper Med.

[25] Beaven A, Sellon E, Ballard M (2021). Combat application tourniquet fares well in a chemical, biological, radiological or nuclear dress state. BMJ Mil Health.

[26] Taylor L, Watkins SL, Marshall H (2016). The impact of different environmental conditions on cognitive function: a focused review. Front Physiol Jan.

[27] Flouris AD, Westwood DA, Cheung SS (2007). Thermal balance effects on vigilance during 2-hour exposures to -20 degrees C. Aviat Space Environ Med.

[28] Teichner WH (1958). Reaction time in the cold. J Appl Psychol.

[29] Muller MD, Gunstad J, Alosco ML (2012). Acute cold exposure and cognitive function: evidence for sustained impairment. Ergonomics.

[30] Davies CT, Young K (1983). Effect of temperature on the contractile properties and muscle power of triceps surae in humans. J Appl Physiol Respir Environ Exerc Physiol Jul.

[31] Havenith G, Heus R, Daanen HA (1995). The hand in the cold, performance and risk. Arct Med Res.

[32] Heus R, Daanen HA, Havenith G (1995). Physiological criteria for functioning of hands in the cold: a review. Appl Ergon Feb.

[33] Cheung SS, Montie DL, White MD (2003). Changes in manual dexterity following short-term hand and forearm immersion in 10 degrees C water. Aviat Space Environ Med.

[34] Cremonini C, Nee N, Demarest M et al. (2021) Evaluation of the efficacy of commercial and noncommercial tourniquets for extremity hemorrhage control in a perfused cadaver model. J Trauma Acute Care Surg. Mar 1;90(3):522–526. 10.1097/TA.0000000000003033. 10.1097/TA.0000000000003033 33230091

[35] Wall PL, Buising CM, Nelms D (2019). Masimo Perfusion Index Versus Doppler for Tourniquet Effectiveness Monitoring. J Spec Oper Med.

[36] Wall PL, Buising CM, Grulke L (2017). Effectiveness of pulse Oximetry Versus Doppler for Tourniquet Monitoring. J Spec Oper Med.

[37] Zeng Z, Centner C, Gollhofer A (2019). Blood-Flow-Restriction training: validity of pulse oximetry to assess arterial occlusion pressure. Int J Sports Physiol Perform Aug.

[38] Lima-Soares F, Pessoa KA, Torres Cabido CE, et al. Determining the arterial occlusion pressure for blood Flow Restriction: pulse oximeter as a New Method compared with a Handheld Doppler. J Strength Cond Res Apr. 2020;29. 10.1519/JSC.000000000000362. 10.1519/JSC.0000000000003628 32379239

[39] Chan ED, Chan MM, Chan MM (2013). Pulse oximetry: understanding its basic principles facilitates appreciation of its limitations. Respir Med.

[40] Schramm WM, Bartunek A, Gilly H. Effect of local limb temperature on pulse oximetry and the plethysmographic pulse wave. Int J Clin Monit Comput. 1997;14(1):17–22. 10.1007/BF03356574. PMID: 9127780. 10.1007/BF03356574 9127780

[41] Ross EM, Matteucci MJ, Shepherd M (2013). Measuring arterial oxygenation in a high altitude field environment: comparing portable pulse oximetry with blood gas analysis. Wilderness Environ Med.

[42] Graham B, Breault MJ, McEwen JA et al. (1993) Occlusion of arterial flow in the extremities at subsystolic pressures through the use of wide tourniquet cuffs. Clin Orthop Relat Res Jan;(286):257–61. 8425355

[43] Wall PL, Sahr SM, Buising CM (2015). Different Width and Tightening System: emergency tourniquets on distal limb segments. J Spec Oper Med.

