# Supplementary material for: Tourniquet self-application assessment in cold weather conditions
Source: BMC Emerg Med. 2023 Aug 31;23:101. doi: 10.1186/s12873-023-00871-1 (PMC10472695; doi:10.1186/s12873-023-00871-1)
Supplement: Supplementary file 1 — Supplementary Material 1 [file 12873_2023_871_MOESM1_ESM.pdf]

POST-ACTIVITY SURVEY ON TOURNIQUETS
Spanish Antarctic Base (BAE) Juan Carlos I

1.- ***Demographics***

1.1 - Age ___________

1.2 – Gender

**M**	**F**

1.3 – What is your training area or task within the Spanish Antarctic Base research team?

____

2. Ease of self-application

Scoring scale: 1 minimum score (less easy)
10 full score (easiest)

2.1 - How do you rate the Combat Application Tourniquet (CAT) self-application technique in terms of its ease?

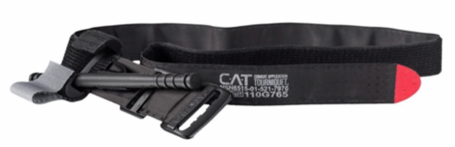

Upper extremity (one handed self-application)

**1**	**2**	**3**	**4**	**5**	**6**	**7**	**8**	**9**	**10**

Lower extremity (two handed self-application

**1**	**2**	**3**	**4**	**5**	**6**	**7**	**8**	**9**	**10**

Explain

____

2.1 - How do you rate the Ratcheting Mechanical Tourniquet (RMT) self-application technique in terms of its ease?

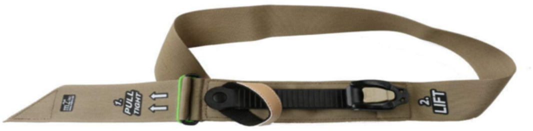

Upper extremity (one handed self-application)

1	2	3	4	5	6	7	8	9	10

Lower extremity (two handed self-application

1	2	3	4	5	6	7	8	9	10

Explain

2.3 - How do you rate the OMNA ratcheting tourniquet self-application technique in terms of its ease?

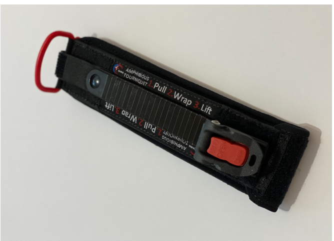

Upper extremity (one handed self-application)

1	2	3	4	5	6	7	8	9	10

Lower extremity (two handed self-application

1	2	3	4	5	6	7	8	9	10

Explain

2.4 - How do you rate the Stretch Wrap and Tuck (SWAT-T) tourniquet self-application technique in terms of its ease?

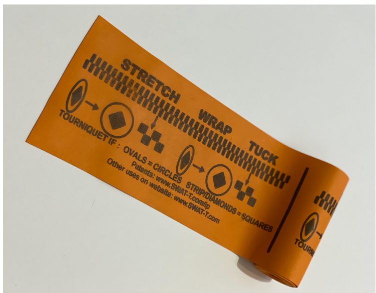

Upper extremity (one handed self-application)

1	2	3	4	5	6	7	8	9	10

Lower extremity (two handed self-application

1	2	3	4	5	6	7	8	9	10

Explain

2.5 - How do you rate the Rapid Application Tourniquet System (RATS) self-application technique in terms of its ease?

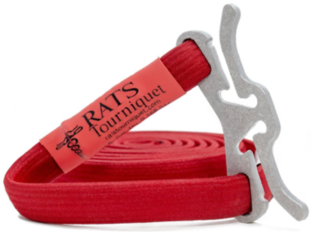

Upper extremity (one handed self-application)

1	2	3	4	5	6	7	8	9	10

Lower extremity (two handed self-application

1	2	3	4	5	6	7	8	9	10

Explain

---

3. Tourniquet tolerance based on pain sensation after self-applied.

Scoring scale: 1 minimum score (less tolerable)
10 full score (most tolerable)

3.1 - How would you rank the tolerance to the Combat Application Tourniquet (CAT) after it is self-applied.

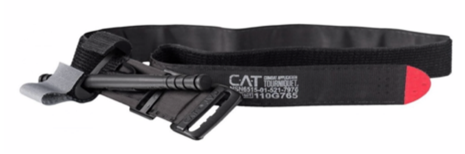

1	2	3	4	5	6	7	8	9	10

3.2 - How would you rank the tolerance to the Ratcheting Mechanical Tourniquet (RMT) tourniquet after it is self-applied.

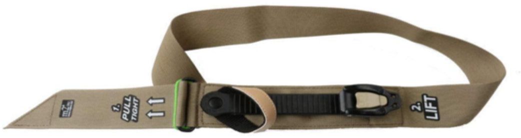

1	2	3	4	5	6	7	8	9	10

3.3 - How would you rank the tolerance to the OMNA ratcheting tourniquet after it is self-applied.

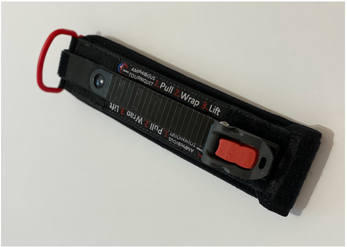

1	2	3	4	5	6	7	8	9	10

3.4 - How would you rank the tolerance to the Stretch Wrap and Tuck (SWAT-T) tourniquet after it is self-applied.

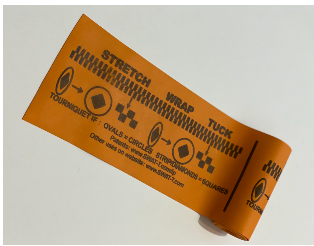

1	2	3	4	5	6	7	8	9	10

3.5 - How would you rank the tolerance to the Rapid Application Tourniquet System (RATS) after it is self-applied.

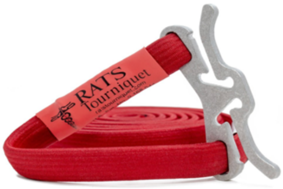

1	2	3	4	5	6	7	8	9	10

4.- INDICATE which of the tested extremity tourniquets would you personally prefer for the field conditions encountered in Antarctica during the field study: CAT / RMT / OMNA / SWAT-T / RATS (choose only one option):

